# Association of Health and Social Spending With Health Outcomes in OECD Countries

**DOI:** 10.1111/1475-6773.14660

**Published:** 2025-07-24

**Authors:** Sungchul Park, Joseph L. Dieleman, Rockli Kim, S. V. Subramanian

**Affiliations:** ^1^ Department of Health Policy and Management, College of Health Science Korea University Seoul Republic of Korea; ^2^ L‐ HOPE Program for Community‐ Based Total Learning, L‐ HOPE Program for Community‐ Based Total Learning Health Systems Korea University Seoul Republic of Korea; ^3^ Institute for Health Metrics and Evaluation University of Washington Seattle Washington USA; ^4^ Department of Social and Behavioral Sciences Harvard T.H. Chan School of Public Health Boston Massachusetts USA

**Keywords:** death, disability‐adjusted life years, health outcomes, health spending, social spending

## Abstract

**Objectives:**

To examine the associations of health and social spending with health outcomes, including Disability‐Adjusted Life Years (DALY), Years of Life Lost (YLL), Years Lived with Disability (YLD), death, and life expectancy at birth among Organization for Economic Cooperation and Development (OECD) member countries from 2000 to 2019.

**Study Setting and Design:**

We conducted a retrospective longitudinal study.

**Data Sources and Analytical Sample:**

Our sample included 36 OECD member countries as of 2019 using data from the Global Burden of Disease Study 2021, the OECD, and the World Bank.

**Principal Findings:**

Fixed‐effect analysis revealed significant associations of health and social spending with health outcomes, but the patterns varied. Specifically, a one‐percentage‐point increase in health spending was associated with a 1.43% (95% CI: −1.86, −1.01) decrease in the death rate per 100,000 population and a 0.68% (0.56, 0.79) increase in YLD per 100,000 population. In contrast, a one‐percentage‐point increase in social spending was associated with a 0.29% (−0.45, −0.12) reduction in DALYs, primarily driven by a 0.30% (−0.37, −0.23) decrease in YLDs and a 0.07% (0.03, 0.12) increase in life expectancy. No significant associations were found for the remaining outcomes. These associations remained robust when incorporating one‐ and two‐year lagged effects.

**Conclusions:**

These findings highlight the distinct mechanisms through which health and social spending impact health outcomes. Health spending predominantly influenced mortality, while social spending was more closely associated with improvements in quality‐of‐life measures. Policymakers should consider these complementary effects when designing interventions to optimize health outcomes.

**Summary**

- What is known on this topic
  ○ Evidence suggests that health and social spending contribute to higher life expectancy and lower mortality.
  ○ However, the mechanisms through which these investments influence both the quality and quantity of life remain less well known.
- What this study adds
  ○ Between 2000 and 2019, higher health spending was associated with lower mortality rates and higher Years Lived with Disability (YLD)
  ○ In contrast, higher social spending was associated with lower Disability‐Adjusted Life Years (DALYs), primarily through declines in YLDs, as well as higher life expectancy.
  ○ These findings suggest distinct pathways through which health and social spending influence health outcomes.
  ○ Health spending was primarily associated with reductions in mortality, whereas social spending was more closely linked to improvements in quality‐of‐life outcomes.

## 1 Introduction

Improving population health is a paramount goal of health systems worldwide. Achieving this objective requires comprehensive system‐level intervention that addresses modifiable factors, including both medical and social risk factors [1, 2]. Health spending primarily targets medical risk factors through disease prevention and management [3]. The definition of social spending varies across contexts and countries, but generally includes services aimed at addressing broader social risk factors such as living conditions and access to essential resources [3].

Both health and social spending are key determinants of health outcomes at both national and subnational levels [3, 4, 5, 6, 7, 8, 9, 10, 11, 12, 13, 14, 15, 16, 17]. Studies have shown that health and social spending contribute to higher life expectancy and lower mortality [3, 4, 5, 6, 7, 8, 9, 18, 19, 20, 21, 22]. However, evidence suggests that medical care alone accounts for only about 10% of premature death, whereas nearly 40% of deaths are attributable to adverse social and environmental [1, 2]. This highlights that social determinants—including income, education, housing, and access to nutritious food—may have a far greater influence on health outcomes [1, 2]. Moreover, meta‐analyses of social interventions indicate significant health benefits from early‐life investments [23].

Despite these findings, much of the existing research has predominantly focused on quantity of life measures, such as mortality and life expectancy, while quality‐of‐life indicators remain underexplored. Evidence suggests a complementary relationship between health and social spending, as countries that invest substantially in one domain tend to allocate more resources to the other [24]. However, the distinct contributions of these spending categories to different health dimensions remain an area requiring further investigation. While health spending can extend life expectancy [6, 18, 19, 20, 21, 22], it may not necessarily improve quality of life [25]. Prolonging life without improving quality may lead to extended periods of morbidity and lower well‐being, ultimately increasing the overall disease burden. In contrast, social spending is also associated with improvements in a range of health outcomes, including mortality, disease prevalence, severe maternal morbidity, and low birth weight [7, 11, 12, 13, 14, 15]. However, social investments appear to exert a stronger influence on overall quality of life [26].

Our study builds upon prior work by examining both the quantity and quality dimensions of health outcomes (Disability‐Adjusted Life Years [DALYs], Years of Life Lost [YLLs], Years Lived with Disability [YLDs], death, and life expectancy at birth) in relation to health and social spending among Organization for Economic Cooperation and Development (OECD) member countries from 2000 to 2019. By incorporating multiple health indicators, our analysis offers a more comprehensive understanding of how spending decisions impact both longevity and well‐being. This nuanced approach provides valuable insights for policymakers seeking to optimize resource allocation to achieve sustainable health improvements.

## 2 Methods

### 2.1 Data

We conducted a retrospective longitudinal study using panel data from the Global Burden of Disease Study 2021 as well as from the OECD and World Bank. The GBD Study quantifies health loss due to various diseases, injuries, and risk factors at global, regional, and national levels. From this dataset, we extracted estimates for DALYs, YLLs, YLDs, death rates, and life expectancy at birth from 2000 to 2019. The OECD Health Expenditure and Financing Database and the OECD Social Expenditure Database provide internationally comparable statistics on health and social spending. From this data, we derived estimates for health and social spending. Finally, we obtained country‐specific characteristics from the OECD, World Bank datasets, and the GBD Study.

### 2.2 Sample

We included 36 OECD member countries as of 2019, most of which are classified as developed nations with high‐income economies. While there were 37 OECD member countries in 2019, Costa Rica was excluded due to incomplete data on health and social spending. These countries were selected for several reasons. First, well‐established national accounting systems in these countries provide reliable and consistent data for health and social spending. In contrast, such data is not available in many low‐ and middle‐income countries. Second, these countries have relatively advanced systems for health and social services, which enable precise estimations of the impacts of health and social spending.

### 2.3 Outcome Variables

We included five outcome measures: DALY, YLL, YLD, death rates per 100,000 population, and life expectancy at birth. DALYs capture both the quantity and quality of care through YLLs and YLDs, respectively. In contrast, death rates and life expectancy reflect only the quantity of life. In the descriptive analysis, outcomes were reported on the original scale. For the regression analysis, all outcome variables were log‐transformed to address skewed distributions.

### 2.4 Independent Variables

Our key independent variables were health and social spending as a percentage of gross domestic product (GDP). Although spending was also examined on a per capita basis, substantial variations in per capita expenditures across countries present challenges for direct comparisons. By contrast, measuring relative spending as a percentage of GDP offers a standardized metric, facilitating more consistent and reliable cross‐country comparisons. Consequently, adjusting for purchasing power parity is less relevant in this context. Health spending was defined as a wide range of expenditures on health care goods and services, which includes personal health care, public health, and preventive services [27]. The OECD Health Expenditure and Financing Database quantifies total health spending by including all costs associated with seeking health care, including mandatory government spending, voluntary health insurance, and private out‐of‐pocket expenses. Social spending was defined as expenditures by countries on programs aimed at supporting the standard of living of disadvantaged or vulnerable groups [28]. The OECD Social Expenditure Database measures total social spending, which includes expenditures on old age, survivors, incapacity‐related benefits, family, active labor‐market programs, unemployment, housing, and other.

While OECD data provide valuable insights, several limitations must be acknowledged, especially for social spending [29]. Specifically, the database has a limited scope and may not fully capture all dimensions of health and social spending. Health spending includes government and household‐funded expenditures but excludes services not covered by the health system. Similarly, social spending classifications can be ambiguous, as expenditures categorized as ‘exclusively private,’ such as out‐of‐pocket health payments, may also function as social spending. Additionally, the classification of social expenditures varies across countries, as the inclusion or exclusion of specific items often depends on data availability and national welfare system differences. The database also does not fully account for public services that contribute to health outcomes, such as education beyond early childhood programs and infrastructure investments. Moreover, private social spending data are reported less systematically than public spending, leading to potential underreporting—especially in countries where private providers, charities, and philanthropic organizations play a significant role. Even within the OECD Social Expenditure Database's defined scope, classification inconsistencies persist. For example, Swiss occupational pensions are classified as mandatory private spending due to legal requirements, while Dutch occupational pensions—despite comparable government involvement—are classified as voluntary. These discrepancies highlight the challenges of using the OECD Social Expenditure Database for standardized cross‐national comparisons.

To adjust for differences in sample characteristics, several covariates were included in the analysis. Demographic factors considered were age distribution and the percentage of females. Socioeconomic differences were assessed using the Socio‐Demographic Index (SDI), which serves as a summary measure of a region's socio‐demographic development based on average income per person, educational attainment, and total fertility rate [30], as well as unemployment rates. Health status differences were captured through the population attributable fraction of modifiable risk factors that were considered not to be driven by the health care sector. These risk factors included environmental risks (e.g., air pollution), occupational risks (e.g., unsafe work environments), and behavioral factors (e.g., smoking and alcohol consumption). This variable represents the proportional reduction in disease or mortality that could be achieved if exposure to a specific risk factor were reduced to an ideal exposure scenario.

### 2.5 Statistical Analysis

We first analyzed trends in outcomes and key independent variables from 2000 to 2019. To assess the association of health and social spending with log‐transformed health outcomes, we employed a fixed effects model, which accounts for unobserved, time‐invariant factors within countries. This approach allows us to control for structural characteristics that do not change over time, such as long‐standing health system frameworks, institutional policies, and cultural attitudes toward health and social spending. By differencing out these time‐invariant factors, the fixed effects model isolates within‐country variations, thereby mitigating the risk of omitted variable bias. This ensures that the estimated associations reflect changes in health and social spending over time within each country rather than differences across countries, potentially improving the internal validity of the analysis. However, we acknowledge that fixed effects models help address potential confounding from unobserved heterogeneity, but do not account for time‐varying confounders that may simultaneously affect both spending and health outcomes. Given this limitation, we also considered using a random effects model. However, random effects models rely on the assumption that unobserved factors are uncorrelated with explanatory variables—an assumption that is unlikely to hold in this context. Consequently, we determined that the fixed effects approach was more appropriate for this analysis. Initially, we performed the model without adjusting for covariates. To address potential confounding from time‐varying factors, we subsequently included the previously mentioned covariates in our analysis. Given that neither the outcomes nor the primary independent variables were age‐adjusted measures, we accounted for these factors as covariates in the model. To assess potential non‐linear relationships between spending measures and health outcomes, we conducted a series of diagnostic tests. First, we regressed the outcome variable on all control variables, excluding health and social spending, and examined the residuals. We then plotted these residuals against health and social spending separately to visually assess the functional form of each relationship. To further evaluate potential deviations from linearity, we included a best‐fit line in the scatter plots to observe overall trends in the data. If the best‐fit line closely followed the distribution of the data points without substantial curvature or deviation, we determined that additional non‐linearity adjustments were unnecessary. This visual assessment suggested a more plausible linear relationship, leading us to adopt a linear regression model. Recognizing that the effects of health and social spending may emerge over time, we analyzed these variables concurrently and with one‐year and two‐year lags to capture potential delayed effects.

To assess the robustness of our findings, we conducted several sensitivity analyses. First, education is a critical social determinant of health, but education spending is not included in the OECD Social Expenditure Database, except for certain childcare and early education services. To address this limitation, we obtained data on education spending from the OECD's educational finance indicators data set. Following prior research [24], we additionally incorporated public and mandatory private education spending into social spending and conducted the analysis with this expanded definition. However, due to missing data for some countries, the results of this analysis may not be directly comparable to those from the original analysis. In addition, we analyzed age‐standardized health outcomes to facilitate more meaningful comparisons, even though age adjustment was already included in the analysis. While the primary models do not explicitly account for reverse causality, we conducted a sensitivity analysis to explore this possibility. In this analysis, health outcomes were used as independent variables with one‐ and two‐year lags, and each spending measure was treated as the outcome variable. The lack of statistically significant associations indicates limited evidence of reverse causality. In all analyses, we included year and country fixed effects, and robust standard errors were used.

## 3 Results

### 3.1 Sample Characteristics

Among the 36 countries studied, the cross‐country mean of certain sample characteristics changed over time (Table 1). The proportion of individuals aged 60–69 increased from 8.8% in 2000 to 10.0% in 2010 and 11.3% in 2019, while the proportion of those aged 70 and older rose from 9.4% in 2000 to 10.8% in 2010 and 12.7% in 2019. Unemployment rates increased from 7.7% in 2000 to 9.1% in 2010 but then decreased to 5.7% in 2019. However, there were small changes in the SDI and population attributable fraction of modifiable risk factors.

**TABLE 1:** Sample characteristics for 2000, 2010, and 2019.

	Mean (SD)		
	2000	2010	2019
Outcomes			
Rates per 100,000 population			
DALY	31,564 (5396)	30,019 (5271)	29,752 (4876)
YLL	19,141 (4911)	17,070 (4795)	16,351 (4348)
YLD	12,424 (1118)	12,949 (1048)	13,401 (1027)
Death	892 (221)	864 (225)	902 (235)
Life expectancy at birth	77 (3)	80 (3)	81 (3)
Primary independent variables			
Spending as a percentage of GDP			
Health spending	7.2 (1.7)	8.7 (2.1)	8.9 (2.3)
Social spending	12.9 (4.6)	15.0 (4.7)	14.6 (4.7)
Covariates			
Age proportions			
10–19	13.9 (2.6)	12.3 (2.3)	11.3 (2.0)
20–29	14.5 (1.7)	13.6 (1.6)	12.5 (1.7)
30–39	15.1 (1.3)	14.3 (1.4)	13.6 (1.1)
40–49	14.0 (1.3)	14.4 (1.4)	13.9 (1.4)
50–59	11.4 (1.9)	12.8 (1.5)	13.4 (1.6)
60–69	8.8 (1.9)	10.0 (1.9)	11.3 (1.6)
70+	9.4 (2.5)	10.8 (2.8)	12.7 (3.2)
Percent of female	44.9 (2.2)	45.4 (2.0)	45.5 (1.8)
SDI index	0.8 (0.1)	0.8 (0.1)	0.8 (0.1)
Unemployment rates	7.7 (4.4)	9.1 (4.3)	5.7 (3.3)
PAF	0.4 (0.0)	0.4 (0.0)	0.4 (0.0)

*Note:* DALY stands for disability‐adjusted life year, YLL refers to years of life lost, and YLD represents years lived with disability. GDP is gross domestic product, SDI is the socio‐demographic index, and PAF denotes population attributable fraction. Health outcomes were reported on the original scale before log transformation. Health spending was defined as a wide range of expenditures on health care goods and services, which includes personal health care, public health, and preventive services. The OECD Health Expenditure and Financing Database quantifies total health spending by including all costs associated with seeking health care, including mandatory government spending, voluntary health insurance, and private out‐of‐pocket expenses. Social spending was defined as expenditures by countries on programs aimed at supporting the standard of living of disadvantaged or vulnerable groups. The OECD Social Expenditure Database measures total social spending, which includes expenditures on active labor‐market programs, incapacity and unemployment benefits, family programs, housing, and pensions. Data from the Global Burden of Disease Study 2021 as well as from the OECD and World Bank.

### 3.2 Trends in Health Outcomes

On average, DALY and YLL rates (per 100,000 population) decreased over time while YLD, death rates (per 100,000 population) and life expectancy at birth increased (Tables 1 and 2). Specifically, DALY rates declined from an average of 31,564 in 2000 to 29,752 in 2019. The most pronounced reductions in DALYs were observed in Estonia, with a 22.3% decrease, and Norway, with a 16.6% decrease. Similarly, YLL rates declined from 19,141 in 2000 to 16,351 in 2019. In contrast, YLD rates increased modestly, rising from 12,424 in 2000 to 13,401 in 2019. Death rates also increased from 892 in 2000 to 902 in 2019. However, substantial country‐specific variations were observed, ranging from a 23.2% decrease in Norway to a 43.9% increase in Japan. Finally, life expectancy at birth increased across all countries, rising from an average of 77.0 years in 2000 to 81.1 years in 2019. The most pronounced gain was observed in Estonia, with an 11.4% increase.

**TABLE 2:** Trends in health outcomes from 2000 to 2019.

Country	DALYs rates per 100,000 population			YLL rates per 100,000 population			YLD rates per 100,000 population			Death rates per 100,000 population			Life expectancy at birth		
	2000	2019	% Change	2000	2019	% Change	2000	2019	% Change	2000	2019	% Change	2000	2019	% Change
Australia	27,007	25,963	−3.9	14,071	12,177	−13.5	12,935	13,786	6.6	679	667	−1.8	79.7	83.2	4.4
Austria	31,308	29,274	−6.5	18,286	15,645	−14.4	13,021	13,630	4.7	954	933	−2.2	78.4	82.1	4.7
Belgium	32,853	30,092	−8.4	19,696	16,246	−17.5	13,157	13,846	5.2	1015	963	−5.1	77.9	81.8	5.0
Canada	26,819	27,978	4.3	14,785	14,333	−3.1	12,034	13,645	13.4	717	770	7.4	79.3	82.3	3.8
Chile	25,058	25,479	1.7	13,966	13,013	−6.8	11,093	12,466	12.4	526	590	12.2	76.9	80.6	4.8
Czechia	35,412	33,374	−5.8	22,771	19,567	−14.1	12,642	13,807	9.2	1075	1062	−1.2	75.1	79.5	5.9
Denmark	33,897	29,404	−13.3	20,833	16,068	−22.9	13,064	13,337	2.1	1074	929	−13.5	77.0	81.5	5.8
Estonia	44,965	34,929	−22.3	31,886	21,211	−33.5	13,079	13,718	4.9	1318	1148	−12.9	70.9	79.0	11.4
Finland	32,440	30,801	−5.1	19,078	16,593	−13.0	13,362	14,208	6.3	952	979	2.8	78.0	82.2	5.4
France	30,267	28,737	−5.1	17,318	14,961	−13.6	12,948	13,777	6.4	880	897	1.9	79.0	83.0	5.1
Germany	33,187	33,190	0.0	19,607	18,384	−6.2	13,580	14,806	9.0	1019	1105	8.4	78.3	81.3	3.8
Greece	31,134	33,762	8.4	17,884	19,293	7.9	13,250	14,469	9.2	934	1206	29.1	78.3	81.2	3.7
Hungary	43,772	39,624	−9.5	30,610	25,879	−15.5	13,162	13,745	4.4	1326	1332	0.5	71.8	76.7	6.8
Iceland	23,797	23,457	−1.4	12,458	11,307	−9.2	11,339	12,150	7.2	631	649	2.9	80.2	83.3	3.9
Ireland	28,722	24,381	−15.1	16,881	11,744	−30.4	11,842	12,638	6.7	810	636	−21.5	76.7	82.3	7.3
Israel	22,945	19,852	−13.5	12,700	9221	−27.4	10,245	10,631	3.8	583	492	−15.6	78.8	83.4	5.8
Italy	31,956	30,499	−4.6	18,116	15,991	−11.7	13,840	14,508	4.8	986	1053	6.8	79.6	83.4	4.8
Japan	26,915	29,810	10.8	14,772	16,005	8.3	12,142	13,805	13.7	748	1076	43.9	81.6	85.0	4.2
Korea	24,074	23,580	−2.1	14,006	11,167	−20.3	10,068	12,414	23.3	520	570	9.6	76.2	83.4	9.4
Latvia	45,685	41,741	−8.6	32,520	27,842	−14.4	13,165	13,899	5.6	1333	1426	7.0	70.6	76.0	7.6
Lithuania	39,971	40,910	2.3	27,312	26,751	−2.1	12,659	14,159	11.8	1106	1372	24.1	72.2	76.4	5.8
Luxembourg	29,513	24,843	−15.8	16,941	12,110	−28.5	12,572	12,734	1.3	838	695	−17.1	78.3	82.7	5.6
Mexico	26,881	28,220	5.0	17,656	17,414	−1.4	9225	10,805	17.1	451	585	29.7	74.0	75.6	2.2
Netherlands	29,118	28,449	−2.3	17,189	15,177	−11.7	11,928	13,271	11.3	882	890	0.9	78.2	82.0	4.9
New Zealand	27,979	26,388	−5.7	15,263	12,938	−15.2	12,716	13,450	5.8	712	679	−4.6	78.5	81.9	4.3
Norway	30,774	25,654	−16.6	17,892	12,668	−29.2	12,882	12,987	0.8	990	760	−23.2	78.8	83.1	5.5
Poland	34,697	34,636	−0.2	22,661	21,382	−5.6	12,035	13,254	10.1	958	1065	11.2	73.9	78.0	5.5
Portugal	34,130	31,896	−6.5	20,681	17,375	−16.0	13,449	14,520	8.0	1000	1052	5.2	76.7	82.0	6.9
Slovak Republic	34,267	33,324	−2.8	22,330	20,364	−8.8	11,937	12,960	8.6	958	988	3.1	73.7	77.7	5.4
Slovenia	33,207	30,907	−6.9	20,496	17,039	−16.9	12,711	13,868	9.1	928	985	6.1	76.3	81.6	6.9
Spain	29,392	28,284	−3.8	16,994	14,628	−13.9	12,398	13,656	10.1	884	913	3.3	79.4	83.2	4.8
Sweden	30,572	27,304	−10.7	17,893	14,070	−21.4	12,679	13,234	4.4	1053	878	−16.6	79.9	83.3	4.3
Switzerland	29,402	25,943	−11.8	15,740	12,009	−23.7	13,662	13,933	2.0	855	753	−11.9	80.3	84.7	5.5
Türkiye	29,758	25,474	−14.4	19,832	14,240	−28.2	9926	11,235	13.2	525	608	15.8	73.2	77.4	5.7
United Kingdom	32,602	29,273	−10.2	19,490	15,611	−19.9	13,113	13,661	4.2	1040	895	−13.9	77.8	81.4	4.6
United States	31,838	33,631	5.6	18,446	18,210	−1.3	13,392	15,421	15.2	850	865	1.8	76.9	79.1	2.9

*Note:* DALY stands for disability‐adjusted life year, YLL refers to years of life lost, and YLD represents years lived with disability. Health outcomes were reported on the original scale before log transformation. Source: Data from the Global Burden of Disease Study 2021.

### 3.3 Trends in Health and Social Spending

Overall, health and social spending increased over time, but substantial variations were observed across countries (Table 3). Specifically, health spending increased from 7.0% of GDP in 2000 to 8.6% of GDP in 2019, while social spending rose from 13.6% of GDP in 2000 to 15.9% of GDP in 2019. In almost all countries, health and social spending increased between 2000 and 2019, while only a few countries experienced a decline. The most significant increases in health spending were in South Korea with a 110.6% increase, followed by Japan, which experienced a 56.0% increase. Similarly, the largest increases in social spending occurred in South Korea with a 108.4% increase and Mexico with a 96.6% increase.

**TABLE 3:** Trends in health and social spending (measured as percentage of Gross Domestic Product) from 2000 to 2019.

Country	Health spending			Social spending		
	2000	2019	% Change	2000	2019	% Change
Australia	7.6	10.2	34.5	15.9	17.8	11.6
Austria	9.2	10.5	14.0	21.3	22.0	3.6
Belgium	8.0	10.8	34.5	19.4	22.3	15.1
Canada	8.3	11.1	34.1	13.9	16.5	18.9
Chile	7.0	9.3	33.0	9.0	9.4	3.9
Czechia	5.7	7.6	33.3	13.1	13.6	4.4
Denmark	8.1	10.2	25.3	22.5	25.3	12.7
Estonia	5.2	6.8	31.2	10.0	13.2	32.3
Finland	7.1	9.2	29.3	19.5	24.4	24.8
France	9.6	11.1	15.9	21.5	24.2	12.8
Germany	9.9	11.7	18.5	20.1	19.8	−1.6
Greece	7.2	8.2	13.3	13.4	20.7	55.3
Hungary	6.8	6.3	−7.4	15.4	13.4	−12.7
Iceland	8.9	8.6	−2.9	12.5	18.5	48.3
Ireland	5.9	6.7	13.9	11.4	8.9	−21.3
Israel	6.6	7.2	9.2	13.6	12.9	−5.0
Italy	7.6	8.7	14.4	18.6	23.0	23.4
Japan	7.0	11.0	56.0	12.8	15.8	24.0
Korea	3.9	8.1	110.6	4.8	9.9	108.4
Latvia	5.4	6.6	22.3	12.6	12.6	0.0
Lithuania	6.2	7.0	12.6	11.2	12.8	14.8
Luxembourg	5.9	5.5	−7.8	13.9	17.9	28.4
Mexico	4.2	5.3	25.0	2.4	4.7	96.6
Netherlands	7.7	10.1	31.5	19.6	20.4	3.8
New Zealand	7.5	9.1	21.4	12.4	16.5	32.4
Norway	7.7	10.4	35.6	18.1	21.3	17.5
Poland	5.3	6.5	22.1	16.5	16.9	2.1
Portugal	8.6	9.5	10.6	13.8	18.0	30.4
Slovak Republic	5.3	6.9	30.6	13.6	12.8	−5.9
Slovenia	7.8	8.5	8.8	16.4	15.3	−6.9
Spain	6.8	9.1	34.4	14.6	18.8	28.6
Sweden	7.3	10.8	47.7	23.2	22.0	−5.3
Switzerland	9.1	11.4	25.2	16.7	19.3	15.5
Turkey	4.6	4.4	−5.2	4.7	9.0	93.2
United Kingdom	7.2	10.0	39.0	18.0	17.3	−3.8
United States	12.5	16.6	32.5	12.4	15.6	25.4

*Note:* Health spending was defined as a wide range of expenditures on health care goods and services, which includes personal health care, public health, and preventive services. The OECD Health Expenditure and Financing Database quantifies total health spending by including all costs associated with seeking health care, including mandatory government spending, voluntary health insurance, and private out‐of‐pocket expenses. Social spending was defined as expenditures by countries on programs aimed at supporting the standard of living of disadvantaged or vulnerable groups. The OECD Social Expenditure Database measures total social spending, which includes expenditures on active labor‐market programs, incapacity and unemployment benefits, family programs, housing, and pensions. Source: Data from the OECD Health Expenditure and Financing Database and the OECD Social Expenditure Database.

### 3.4 Association of Health and Social Spending With Health Outcomes

Our fixed‐effect analysis revealed significant associations of health and social spending with health outcomes, but the patterns varied (Table 4). Although significant associations of health and social spending were observed in all health outcomes (eTable A), some of these associations became non‐significant after adjusting for covariates. Specifically, a one‐percentage‐point increase in health spending was associated with a 1.43% (95% CI: −1.86, −1.01) decrease in the death rate per 100,000 population and a 0.68% (95% CI: 0.56, 0.79) increase in YLD per 100,000 population. However, no statistically significant associations were observed between health spending and DALYs, YLLs, or life expectancy. In contrast, a one‐percentage‐point increase in social spending was associated with a 0.29% (95% CI: −0.45, −0.12) decrease in DALYs, a 0.30% (95% CI: −0.37, −0.23) decrease in YLDs, and a 0.07% (95% CI: 0.03, 0.12) increase in life expectancy. No significant associations were found for death rates or YLLs. These findings remained relatively consistent even after incorporating education spending into the social spending category (eTable B). Furthermore, the results were similar when age‐standardized health outcomes were used (eTable C). Full regression results after covariate adjustment are available in eTable D. Our sensitivity analysis found limited evidence of reverse causality, indicating that prior‐year health outcomes were not significantly associated with current‐year health or social spending (eTables E and F).

**TABLE 4:** Associations of health and social spending (measured as percentage of Gross Domestic Product) with log‐transformed health outcomes.

	Estimates (95% CI)				
	DALY rates per 100,000 population	YLL rates per 100,000 population	YLD rates per 100,000 population	Death rates per 100,000 population	Life expectancy at birth
Concurrent					
Health spending	0.23 (−0.04 to 0.5)	−0.42 (−0.84 to 0.01)	0.68 (0.56–0.79)***	−1.43 (−1.86 to −1.01)***	0.03 (−0.04 to 0.1)
Social spending	−0.29 (−0.46 to −0.12)**	−0.22 (−0.49 to 0.05)	−0.3 (−0.37 to −0.23)***	−0.09 (−0.36 to 0.18)	0.07 (0.03–0.12)**
1 year lagged					
Health spending	0.18 (−0.08 to 0.45)	−0.5 (−0.92 to −0.08)*	0.66 (0.55–0.77)***	−1.44 (−1.86 to −1.03)***	0.03 (−0.04 to 0.1)
Social spending	−0.34 (−0.51 to −0.16)***	−0.28 (−0.57 to −0.01)*	−0.29 (−0.37 to −0.22)***	0.01 (−0.27 to 0.29)	0.07 (0.02–0.11)**
2 year lagged					
Health spending	0.2 (−0.05 to 0.45)	−0.47 (−0.87 to −0.06)*	0.65 (0.54–0.76)***	−1.34 (−1.74 to −0.93)***	0.01 (−0.05 to 0.08)
Social spending	−0.24 (−0.4 to −0.08)**	−0.17 (−0.42 to 0.08)	−0.23 (−0.3 to −0.16)***	0.22 (−0.03 to 0.47)***	0.02 (−0.02 to 0.06)

*Note:* Each reported value represents the estimated coefficient multiplied by 100 and can be interpreted as the percent change in health outcomes associated with a one percentage point change in the independent variable. Fixed‐effects models were performed with health spending and social spending as key independent variables and DALY, YLL, YLD, death rates, or life expectancy at birth as the outcome variable. Covariate adjustments accounted for age distribution (percentages in the categories: < 10, 10–19, 20–29, 30–39, 40–49, 50–59, 60–69, and 70+), percentage of females, socio‐demographic index (SDI), unemployment rates, population attributable fraction (PAF), and year. In all analyses, we included year and country fixed effects, and robust standard errors were used. Source: Data from the Global Burden of Disease Study 2021 as well as from the OECD and World Bank.

* *p* < 0.05.

** *p* < 0.01.

*** *p* < 0.001.

These associations remained robust after incorporating one‐ and two‐year lagged effects (Table 4). Specifically, a one‐percentage‐point increase in health spending was consistently associated with reductions in death rates of −1.43% (95% CI: −1.86, −1.01) in the concurrent model, −1.44% (95% CI: −1.86, −1.03) with a one‐year lag, and −1.34% (95% CI: −1.74, −0.93) with a two‐year lag. In addition, a one‐percentage‐point increase in health spending was associated with modest but statistically significant increases in YLDs of 0.68% (95% CI: 0.56, 0.79) in the same year, 0.66% (95% CI: 0.55, 0.77) with a one‐year lag, and 0.65% (95% CI: 0.54, 0.76) with a two‐year lag. In contrast, higher social spending was associated with significantly lower rates of both DALYs and YLDs across all time points. A one‐percentage‐point increase in social spending was associated with reductions in DALYs of −0.29% (95% CI: −0.46, −0.12) in the same year, −0.34% (95% CI: −0.51, −0.16) with a one‐year lag, and −0.24% (95% CI: −0.40, −0.08) with a two‐year lag. Furthermore, a one‐percentage‐point increase in social spending was associated with reductions in YLDs of −0.3% (95% CI: −0.37, −0.23) in the concurrent model, −0.29% (95% CI: −0.37, −0.22) with a one‐year lag, and −0.23% (95% CI: −0.3, −0.16) with a two‐year lag. Social spending was also positively associated with life expectancy, with estimated increases of 0.07% (95% CI: 0.03, 0.12) in the concurrent model and 0.07% (95% CI: 0.02, 0.11) with a one‐year lag. However, the two‐year lag association was not statistically significant. Full regression results after covariate adjustment are presented in eTables G, H.

## 4 Discussion

Using data from 2000 to 2019 across 36 OECD member countries, our analysis indicates that higher health spending was associated with lower death rates but also with higher YLD rates. This finding suggests that while health spending may contribute to extended life expectancy, it could also be correlated with an increased burden of disease. One possible explanation for this is that health spending equips individuals to live longer, but as they do, they accumulate more years lived with disability. Conversely, higher social spending was not associated with changes in mortality but was significantly associated with lower DALY rates, primarily due to reductions in YLD rates, and modest improvements in life expectancy. This finding implies that social spending may be associated with improvements in population health by alleviating the burden of disease and improving quality of life, though its relationship with overall death rates remains modest.

Our findings on the association between health spending and health outcomes are consistent with previous research [6, 18, 19, 20, 21, 22]. Evidence suggests that higher health spending contributes to gains in quality‐adjusted life expectancy or reductions in DALYs [25, 31, 32, 33, 34, 35, 36, 37, 38]. While this may be primarily driven by lower mortality rates, it does not necessarily translate into overall improvements in quality of life [25]. This aligns with our observation that higher health spending is associated with extended life expectancy, but it may not always enhance health‐related quality of life. Notably, higher health spending was associated with an increase in YLD. This suggests that increased health spending can facilitate access to advanced treatments that improve survival rates for serious conditions but may not fully restore health. Consequently, individuals may live longer with functional limitations or disabilities related to their conditions, leading to both extended life expectancy and increased YLD. Furthermore, these associations persist over time, suggesting that the relationship between health spending and outcomes remains consistent in the years following the initial observations. This may be attributed to a portion of health spending being allocated to the management of chronic conditions, which has the potential to improve health outcomes gradually over time.

Our analysis provides further insight into the association between social spending and health outcomes, an area with previously mixed evidence. While some studies focusing on high‐income countries suggest that higher social spending is associated with lower mortality rates, others have found no significant association [3, 4, 5, 6, 7, 8, 9, 10]. Our findings contribute a more nuanced perspective to this debate. Specifically, we observed that higher social spending was significantly associated with reductions in YLD and modest increases in life expectancy. This finding suggests that social spending may be particularly effective in targeting preventable sources of disability—such as those related to poverty, inadequate housing, and limited access to education or employment opportunities. By addressing these social determinants of health, social investment may indirectly enhance quality of life, even if its direct relationship with mortality remains limited. However, the observed effect sizes were smaller than anticipated. Several explanations are possible. First, the measure of social spending used in this study may not fully reflect the range of services and investments that influence health. Second, many social programs are not specifically structured to improve health outcomes, which may limit the extent to which program impacts are detectable in health‐related analyses.

Our findings highlight important policy implications, emphasizing the distinct roles of health and social spending in improving health outcomes. While health spending is essential for reducing mortality through effective disease treatment, social spending plays a critical role in enhancing quality of life by addressing social determinants and preventing disabilities. Given that each country faces unique health priorities and socioeconomic conditions, understanding these differential associations is crucial for effectively enhancing health outcomes. To maximize the impact of both types of spending, it is essential to integrate health and social policies into a cohesive framework [39]. This approach recognizes that health outcomes are influenced not only by health care services but also by broader social factors. Aligning health initiatives with social policy goals can enable governments to create environments that support both extended life expectancy and improved quality of life.

Our study has several limitations. First, our analysis focused exclusively on OECD member countries, which are predominantly high‐income nations. Thus, the findings may not be applicable to other contexts, such as middle‐income or low‐income countries. Second, our measurement of health and social spending relied on data from the OECD, which has its limitations. The OECD notes that inconsistencies in data reporting across countries may reflect variations in local data collection and accounting practices. Third, while we adjusted for several potential confounders, concerns about unmeasured confounding remain in observational analyses. The fixed effect model helps mitigate confounding from unmeasured time‐constant factors. However, confounding from unmeasured time‐varying factors is still a significant issue. Therefore, our findings should not be interpreted as inferring a causal relationship. Finally, the relationship of health and social spending with health outcomes may be bidirectional. While spending can influence health outcomes, health outcomes may also impact spending levels. Our use of lagged effects suggests that reverse causality is unlikely to be the primary driver of our findings, as the results remain consistent. However, some bias may still exist. For example, higher mortality leads to lower health and social spending, while a greater disease burden increases spending. These opposing effects could introduce complexities in causal interpretation.

## 5 Conclusion

This study underscores the differential associations of health and social spending with health outcomes in OECD member countries, showing that health spending was associated with lower mortality, while social spending was primairly associated with lower DALY rates, largely driven by decreases in YLD rates. Policymakers can leverage these insights to develop balanced strategies that ensure health system investments are complemented by robust health care and social policies. An integrated approach to health and social spending may provide a more effective framework for achieving sustainable health improvements, enhancing both the quantity and quality of life.

## Conflicts of Interest

The authors declare no conflicts of interest.

## Supporting information


**Appendix S1.** Supporting Information.

## Data Availability

The data that support the findings of this study are available from the corresponding author upon reasonable request.
