# Supplementary material for: Association of Health and Social Spending With Health Outcomes in OECD Countries
Source: Health Serv Res. 2025 Jul 24;60(6):e14660. doi: 10.1111/1475-6773.14660 (PMC12636253; doi:10.1111/1475-6773.14660)
Supplement: Supplementary file 1 — Appendix S1. Supporting Information. [file HESR-60-e14660-s001.docx]

**Appendix**

**eTable A. Associations of social spending and health spending (measured as percentage of Gross Domestic Product) with health outcomes (measured as rates per 100,000 population) (without covariate adjustment).**

**eTable B. Associations of social spending (after additionally including education spending) and health spending (measured as percentage of Gross Domestic Product) with health outcomes (measured as rates per 100,000 population).**

**eTable C. Associations of social spending and health spending (measured as percentage of Gross Domestic Product) with age-standardized log-transformed health outcomes**

**eTable D. Full regression results for the analysis using concurrent spending measures as the key independent variables.**

**eTable E. Sensitivity analysis for assessing reverse causality in health spending.**

**eTable F. Sensitivity analysis for assessing reverse causality in social spending**.

**eTable G. Full regression results for the analysis using one-year lagged spending measures as the key independent variables.**

**eTable H. Full regression results for the analysis using two-year lagged spending measures as the key independent variables.**

**eTable A. Associations of health and social spending (measured as percentage of Gross Domestic Product) with log-transformed health outcomes (without covariate adjustment).**

|  | Estimates (95% CI) |  |  |  |  |
| --- | --- | --- | --- | --- | --- |
|  | DALY rate | YLL rate | YLD rate | Death rate | Life expectancy |
| All |  |  |  |  |  |
| Concurrent |  |  |  |  |  |
| Health spending | -1.02 (-1.38 to -0.66) | -3.31 (-3.95 to -2.67) | 1.75 (1.51 to 1.99) | -1.06 (-1.61 to -0.5) | 1.03 (0.88 to 1.18) |
| Social spending | -0.47 (-0.67 to -0.26) | -0.85 (-1.21 to -0.49) | 0.2 (0.06 to 0.33) | 0.56 (0.25 to 0.88) | 0.28 (0.19 to 0.37) |
| One-year lagged |  |  |  |  |  |
| Health spending | -0.98 (-1.33 to -0.62) | -3.19 (-3.81 to -2.56) | 1.66 (1.42 to 1.89) | -1.03 (-1.58 to -0.47) | 0.98 (0.83 to 1.12) |
| Social spending | -0.43 (-0.63 to -0.23) | -0.78 (-1.14 to -0.43) | 0.2 (0.07 to 0.33) | 0.68 (0.37 to 1) | 0.28 (0.19 to 0.36) |
| Two-year lagged |  |  |  |  |  |
| Health spending | -0.92 (-1.27 to -0.57) | -3.03 (-3.64 to -2.42) | 1.57 (1.34 to 1.8) | -0.94 (-1.5 to -0.39) | 0.92 (0.78 to 1.07) |
| Social spending | -0.35 (-0.54 to -0.15) | -0.66 (-1 to -0.32) | 0.22 (0.09 to 0.35) | 0.85 (0.54 to 1.16) | 0.27 (0.19 to 0.35) |
| Male |  |  |  |  |  |
| Concurrent |  |  |  |  |  |
| Health spending | -1.51 (-1.98 to -1.04) | -3.74 (-4.48 to -3.01) | 1.95 (1.7 to 2.2) | -1.09 (-1.65 to -0.53) | 1.23 (1.04 to 1.42) |
| Social spending | -0.73 (-1 to -0.46) | -1.08 (-1.5 to -0.66) | 0.12 (-0.02 to 0.26) | 0.25 (-0.07 to 0.57) | 0.35 (0.24 to 0.45) |
| One-year lagged |  |  |  |  |  |
| Health spending | -1.43 (-1.9 to -0.96) | -3.57 (-4.29 to -2.85) | 1.84 (1.6 to 2.09) | -0.98 (-1.54 to -0.43) | 1.16 (0.98 to 1.35) |
| Social spending | -0.71 (-0.98 to -0.45) | -1.05 (-1.46 to -0.64) | 0.13 (-0.01 to 0.27) | 0.34 (0.02 to 0.66) | 0.35 (0.25 to 0.46) |
| Two-year lagged |  |  |  |  |  |
| Health spending | -1.36 (-1.82 to -0.9) | -3.4 (-4.11 to -2.7) | 1.74 (1.5 to 1.99) | -0.88 (-1.44 to -0.32) | 1.1 (0.92 to 1.28) |
| Social spending | -0.62 (-0.88 to -0.37) | -0.94 (-1.33 to -0.54) | 0.15 (0.01 to 0.28) | 0.49 (0.18 to 0.81) | 0.34 (0.24 to 0.44) |
| Female |  |  |  |  |  |
| Concurrent |  |  |  |  |  |
| Health spending | -0.5 (-0.78 to -0.21) | -2.76 (-3.32 to -2.2) | 1.63 (1.39 to 1.87) | -1.02 (-1.63 to -0.4) | 0.86 (0.74 to 0.98) |
| Social spending | -0.17 (-0.33 to -0.01) | -0.51 (-0.83 to -0.19) | 0.24 (0.1 to 0.37) | 0.91 (0.56 to 1.26) | 0.2 (0.14 to 0.27) |
| One-year lagged |  |  |  |  |  |
| Health spending | -0.51 (-0.78 to -0.23) | -2.71 (-3.25 to -2.18) | 1.55 (1.32 to 1.79) | -1.07 (-1.69 to -0.45) | 0.82 (0.71 to 0.94) |
| Social spending | -0.11 (-0.27 to 0.04) | -0.38 (-0.69 to -0.08) | 0.23 (0.1 to 0.37) | 1.06 (0.71 to 1.41) | 0.2 (0.13 to 0.26) |
| Two-year lagged |  |  |  |  |  |
| Health spending | -0.46 (-0.73 to -0.18) | -2.56 (-3.08 to -2.04) | 1.48 (1.25 to 1.71) | -1 (-1.63 to -0.38) | 0.77 (0.66 to 0.89) |
| Social spending | -0.04 (-0.19 to 0.12) | -0.24 (-0.53 to 0.05) | 0.25 (0.12 to 0.38) | 1.23 (0.88 to 1.58) | 0.19 (0.12 to 0.25) |

**eTable B. Associations of social spending (after additionally including education spending) and health spending (measured as percentage of Gross Domestic Product) with log-transformed health outcomes.**

|  | Estimates (95% CI) |  |  |  |  |
| --- | --- | --- | --- | --- | --- |
|  | DALY rate | YLL rate | YLD rate | Death rate | Life expectancy |
| All |  |  |  |  |  |
| Concurrent |  |  |  |  |  |
| Health spending | 0.32 (-0.02 to 0.66) | -0.12 (-0.65 to 0.4) | 0.69 (0.52 to 0.86) | -0.98 (-1.52 to -0.45) | -0.04 (-0.12 to 0.04) |
| Social spending | -0.22 (-0.39 to -0.05) | -0.16 (-0.42 to 0.1) | -0.23 (-0.32 to -0.15) | -0.14 (-0.4 to 0.13) | 0.06 (0.01 to 0.1) |
| One-year lagged |  |  |  |  |  |
| Health spending | 0.42 (0.05 to 0.78) | -0.04 (-0.61 to 0.53) | 0.73 (0.56 to 0.9) | -0.9 (-1.46 to -0.35) | -0.07 (-0.16 to 0.02) |
| Social spending | -0.37 (-0.55 to -0.19) | -0.39 (-0.68 to -0.1) | -0.23 (-0.32 to -0.15) | -0.21 (-0.49 to 0.07) | 0.08 (0.04 to 0.13) |
| Two-year lagged |  |  |  |  |  |
| Health spending | 0.44 (0.05 to 0.83) | 0.03 (-0.59 to 0.65) | 0.71 (0.55 to 0.88) | -0.82 (-1.4 to -0.24) | -0.08 (-0.17 to 0.01) |
| Social spending | -0.24 (-0.43 to -0.05) | -0.22 (-0.52 to 0.08) | -0.21 (-0.3 to -0.13) | -0.05 (-0.33 to 0.23) | 0.04 (0 to 0.09) |

**eTable C. Associations of social spending and health spending (measured as percentage of Gross Domestic Product) with age-standardized log-transformed health outcomes**

|  | Estimates (95% CI) |  |  |  |  |
| --- | --- | --- | --- | --- | --- |
|  | DALY rates per 100,000 population | YLL rates per 100,000 population | YLD rates per 100,000 population | Death rates per 100,000 population | Life expectancy at birth |
| Concurrent |  |  |  |  |  |
| Health spending | 0.58 (0.27 to 0.89) | -0.12 (-0.61 to 0.37) | 0.86 (0.74 to 0.98) | -0.75 (-1.17 to -0.32) | -0.01 (-0.08 to 0.07) |
| Social spending | -0.47 (-0.66 to -0.28) | -0.48 (-0.78 to -0.18) | -0.32 (-0.39 to -0.25) | -0.48 (-0.74 to -0.22) | 0.08 (0.04 to 0.12) |
| One-year lagged |  |  |  |  |  |
| Health spending | 0.54 (0.23 to 0.84) | -0.23 (-0.72 to 0.26) | 0.85 (0.73 to 0.97) | -0.78 (-1.21 to -0.36) | 0 (-0.07 to 0.07) |
| Social spending | -0.5 (-0.7 to -0.3) | -0.49 (-0.8 to -0.17) | -0.33 (-0.41 to -0.26) | -0.39 (-0.66 to -0.12) | 0.08 (0.03 to 0.13) |
| Two-year lagged |  |  |  |  |  |
| Health spending | 0.57 (0.27 to 0.87) | -0.17 (-0.64 to 0.31) | 0.83 (0.72 to 0.95) | -0.69 (-1.1 to -0.27) | -0.02 (-0.09 to 0.05) |
| Social spending | -0.33 (-0.51 to -0.15) | -0.24 (-0.52 to 0.05) | -0.28 (-0.35 to -0.21) | -0.1 (-0.34 to 0.15) | 0.04 (0 to 0.08) |

**eTable D. Full regression results for the analysis using concurrent spending measures as the key independent variables.**

|  | Estimates (95% CI) |  |  |  |  |
| --- | --- | --- | --- | --- | --- |
| Characteristics | DALY | YLL | YLD | Death | Life expectancy |
| Social spending (concurrent) | -0.0029 (-0.0046 to -0.0012) | -0.0022 (-0.0049 to 0.0005) | -0.003 (-0.0037 to -0.0023) | -0.0009 (-0.0036 to 0.0018) | 0.0007 (0.0003 to 0.0012) |
| Health spending (concurrent) | 0.0023 (-0.0004 to 0.005) | -0.0042 (-0.0084 to 0.0001) | 0.0068 (0.0056 to 0.0079) | -0.0143 (-0.0186 to -0.0101) | 0.0003 (-0.0004 to 0.001) |
| Age proportions |  |  |  |  |  |
| 10-19 | 0.0048 (-0.0021 to 0.0117) | -0.0063 (-0.0172 to 0.0046) | 0.0093 (0.0064 to 0.0123) | 0.0165 (0.0055 to 0.0274) | -0.0051 (-0.0069 to -0.0033) |
| 20-29 | -0.004 (-0.0098 to 0.0018) | -0.0168 (-0.026 to -0.0077) | 0.0083 (0.0058 to 0.0107) | -0.0019 (-0.0111 to 0.0073) | -0.0017 (-0.0032 to -0.0002) |
| 30-39 | -0.0006 (-0.0071 to 0.0058) | -0.0152 (-0.0254 to -0.0051) | 0.0119 (0.0091 to 0.0146) | 0.0057 (-0.0046 to 0.0159) | -0.0021 (-0.0038 to -0.0005) |
| 40-49 | -0.0036 (-0.01 to 0.0028) | -0.0209 (-0.0309 to -0.0108) | 0.0127 (0.01 to 0.0154) | 0.0047 (-0.0054 to 0.0148) | -0.0009 (-0.0025 to 0.0008) |
| 50-59 | 0.0022 (-0.0041 to 0.0085) | -0.0148 (-0.0247 to -0.0049) | 0.0168 (0.0141 to 0.0195) | 0.0098 (-0.0002 to 0.0197) | 0.0003 (-0.0014 to 0.0019) |
| 60-69 | 0.0038 (-0.0028 to 0.0104) | -0.0116 (-0.0221 to -0.0012) | 0.0183 (0.0155 to 0.0212) | 0.0152 (0.0047 to 0.0257) | 0.0008 (-0.001 to 0.0025) |
| 70+ | 0.012 (0.0046 to 0.0193) | 0.0031 (-0.0086 to 0.0147) | 0.0213 (0.0182 to 0.0245) | 0.0424 (0.0308 to 0.0541) | 0.0008 (-0.0011 to 0.0027) |
| Percent of female | 0.0336 (0.0223 to 0.045) | 0.0628 (0.0449 to 0.0807) | -0.0046 (-0.0095 to 0.0002) | 0.0214 (0.0034 to 0.0394) | -0.0037 (-0.0066 to -0.0008) |
| SDI index | 0 (0 to 0) | 0 (0 to 0) | 0 (0 to 0) | 0 (0 to 0) | 0 (0 to 0) |
| Unemployment rates | -1.07 (-1.3721 to -0.768) | -1.5449 (-2.0216 to -1.0682) | 0.1458 (0.0172 to 0.2744) | 0.5905 (0.1112 to 1.0699) | 0.1198 (0.0414 to 0.1982) |
| PAF | -0.0004 (-0.0012 to 0.0003) | -0.0015 (-0.0027 to -0.0003) | 0.0007 (0.0003 to 0.001) | -0.001 (-0.0022 to 0.0002) | 0 (-0.0002 to 0.0002) |

Abbreviation: SDI, socio-demographic Index; PAF, population attributable population.

**eTable E. Sensitivity analysis for assessing reverse causality in health spending**.

|  | Estimates (95% CI) |  |  |  |  |
| --- | --- | --- | --- | --- | --- |
| Characteristics | Model 1 | Model 2 | Model 3 | Model 4 | Model 5 |
| DALY |  |  |  |  |  |
| One-year lagged | 2.64 (-1.73 to 7.01) |  |  |  |  |
| Two -year lagged | 0.31 (-3.77 to 4.39) |  |  |  |  |
| YLL |  |  |  |  |  |
| One-year lagged |  | -0.21 (-2.88 to 2.47) |  |  |  |
| Two -year lagged |  | -0.45 (-2.96 to 2.07) |  |  |  |
| YLD |  |  |  |  |  |
| One-year lagged |  |  | 19.27 (1.21 to 37.32) |  |  |
| Two -year lagged |  |  | 2.28 (-15.27 to 19.83) |  |  |
| Death |  |  |  |  |  |
| One-year lagged |  |  |  | -1.33 (-4.37 to 1.72) |  |
| Two -year lagged |  |  |  | -2.07 (-4.95 to 0.8) |  |
| Life expectancy |  |  |  |  |  |
| One-year lagged |  |  |  |  | -4.92 (-23.05 to 13.21) |
| Two -year lagged |  |  |  |  | 3.22 (-14.25 to 20.69) |

Covariate adjustments accounted for age distribution (percentages in the categories: <10, 10–19, 20–29, 30–39, 40–49, 50–59, 60–69, 70+), percentage of females, socio-demographic index (SDI), unemployment rates, population attributable fraction (PAF), and year. In all analyses, we included year and country fixed-effects and robust standard errors were used.

**eTable F. Sensitivity analysis for assessing reverse causality in social spending**.

|  | Estimates (95% CI) |  |  |  |  |
| --- | --- | --- | --- | --- | --- |
| Characteristics | Model 1 | Model 2 | Model 3 | Model 4 | Model 5 |
| DALY |  |  |  |  |  |
| One-year lagged | -4.68 (-11.82 to 2.46) |  |  |  |  |
| Two -year lagged | -9.57 (-37.96 to 18.81) |  |  |  |  |
| YLL |  |  |  |  |  |
| One-year lagged |  | -1.87 (-6.23 to 2.49) |  |  |  |
| Two -year lagged |  | 1.08 (-3.02 to 5.18) |  |  |  |
| YLD |  |  |  |  |  |
| One-year lagged |  |  | -12.52 (-43.27 to 18.22) |  |  |
| Two -year lagged |  |  | 1.08 (-3.02 to 5.18) |  |  |
| Death |  |  |  |  |  |
| One-year lagged |  |  |  | -1.73 (-6.78 to 3.33) |  |
| Two -year lagged |  |  |  | 0.03 (-4.75 to 4.8) |  |
| Life expectancy |  |  |  |  |  |
| One-year lagged |  |  |  |  | 23.21 (-6.25 to 52.67) |
| Two -year lagged |  |  |  |  | -9.57 (-37.96 to 18.81) |

Covariate adjustments accounted for age distribution (percentages in the categories: <10, 10–19, 20–29, 30–39, 40–49, 50–59, 60–69, 70+), percentage of females, socio-demographic index (SDI), unemployment rates, population attributable fraction (PAF), and year. In all analyses, we included year and country fixed-effects and robust standard errors were used.

**eTable G. Full regression results for the analysis using one-year lagged spending measures as the key independent variables.**

|  | Estimates (95% CI) |  |  |  |  |
| --- | --- | --- | --- | --- | --- |
| Characteristics | DALY | YLL | YLD | Death | Life expectancy |
| Social spending (one-year lagged) | -0.0034 (-0.0051 to -0.0016) | -0.0028 (-0.0057 to 0) | -0.0029 (-0.0037 to -0.0022) | 0.0001 (-0.0027 to 0.0029) | 0.0007 (0.0002 to 0.0011) |
| Health spending (one-year lagged) | 0.0018 (-0.0008 to 0.0045) | -0.005 (-0.0092 to -0.0008) | 0.0066 (0.0055 to 0.0077) | -0.0144 (-0.0186 to -0.0103) | 0.0003 (-0.0004 to 0.001) |
| Age proportions |  |  |  |  |  |
| 10-19 | 0.007 (0.0001 to 0.0139) | -0.0037 (-0.0146 to 0.0073) | 0.009 (0.0061 to 0.0119) | 0.0189 (0.008 to 0.0298) | -0.0057 (-0.0075 to -0.0039) |
| 20-29 | -0.0029 (-0.0087 to 0.0028) | -0.0158 (-0.0249 to -0.0066) | 0.0079 (0.0055 to 0.0104) | -0.0019 (-0.011 to 0.0072) | -0.002 (-0.0034 to -0.0005) |
| 30-39 | 0.0007 (-0.0057 to 0.0072) | -0.0134 (-0.0236 to -0.0032) | 0.011 (0.0083 to 0.0138) | 0.0069 (-0.0032 to 0.0171) | -0.0026 (-0.0042 to -0.0009) |
| 40-49 | -0.0026 (-0.009 to 0.0038) | -0.0198 (-0.03 to -0.0096) | 0.0122 (0.0095 to 0.0149) | 0.0055 (-0.0047 to 0.0157) | -0.0012 (-0.0029 to 0.0004) |
| 50-59 | 0.0035 (-0.0028 to 0.0098) | -0.0133 (-0.0233 to -0.0034) | 0.0164 (0.0137 to 0.019) | 0.0113 (0.0014 to 0.0213) | -0.0002 (-0.0018 to 0.0015) |
| 60-69 | 0.0049 (-0.0017 to 0.0116) | -0.0106 (-0.0211 to -0.0001) | 0.0181 (0.0153 to 0.0209) | 0.0149 (0.0044 to 0.0254) | 0.0006 (-0.0011 to 0.0023) |
| 70+ | 0.0128 (0.0055 to 0.0202) | 0.004 (-0.0076 to 0.0156) | 0.0208 (0.0177 to 0.0239) | 0.0418 (0.0302 to 0.0534) | 0.0007 (-0.0012 to 0.0026) |
| Percent of female | 0.0329 (0.0216 to 0.0442) | 0.0624 (0.0444 to 0.0803) | -0.0037 (-0.0085 to 0.0011) | 0.0213 (0.0034 to 0.0392) | -0.0036 (-0.0065 to -0.0007) |
| SDI index | 0 (0 to 0) | 0 (0 to 0) | 0 (0 to 0) | 0 (0 to 0) | 0 (0 to 0) |
| Unemployment rates | -0.9707 (-1.2721 to -0.6694) | -1.4164 (-1.8954 to -0.9374) | 0.1597 (0.0317 to 0.2876) | 0.6906 (0.2128 to 1.1685) | 0.1 (0.022 to 0.1779) |
| PAF | -0.0003 (-0.0011 to 0.0005) | -0.0012 (-0.0025 to 0) | 0.0005 (0.0002 to 0.0009) | -0.0013 (-0.0025 to 0) | 0 (-0.0002 to 0.0002) |

Abbreviation: SDI, socio-demographic Index; PAF, population attributable population.

**eTable H. Full regression results for the analysis using two-year lagged spending measures as the key independent variables.**

|  | Estimates (95% CI) |  |  |  |  |
| --- | --- | --- | --- | --- | --- |
| Characteristics | DALY | YLL | YLD | Death | Life expectancy |
| Social spending (two-year lagged) | -0.0024 (-0.004 to -0.0008) | -0.0017 (-0.0042 to 0.0008) | -0.0023 (-0.003 to -0.0016) | 0.0022 (-0.0003 to 0.0047) | 0.0002 (-0.0002 to 0.0006) |
| Health spending (two -year lagged) | 0.002 (-0.0005 to 0.0045) | -0.0047 (-0.0087 to -0.0006) | 0.0065 (0.0054 to 0.0076) | -0.0134 (-0.0174 to -0.0093) | 0.0001 (-0.0005 to 0.0008) |
| Age proportions |  |  |  |  |  |
| 10-19 | 0.0083 (0.0016 to 0.0151) | -0.0022 (-0.013 to 0.0086) | 0.0086 (0.0057 to 0.0116) | 0.02 (0.0092 to 0.0308) | -0.0061 (-0.0078 to -0.0043) |
| 20-29 | -0.0024 (-0.008 to 0.0033) | -0.0158 (-0.0249 to -0.0067) | 0.0079 (0.0054 to 0.0103) | -0.0028 (-0.0119 to 0.0062) | -0.0021 (-0.0036 to -0.0007) |
| 30-39 | 0.0013 (-0.0051 to 0.0076) | -0.0128 (-0.023 to -0.0026) | 0.0104 (0.0076 to 0.0131) | 0.007 (-0.0031 to 0.0172) | -0.0028 (-0.0044 to -0.0012) |
| 40-49 | -0.0018 (-0.0081 to 0.0046) | -0.0192 (-0.0295 to -0.009) | 0.0119 (0.0091 to 0.0147) | 0.0058 (-0.0044 to 0.016) | -0.0016 (-0.0033 to 0) |
| 50-59 | 0.0046 (-0.0017 to 0.0108) | -0.0123 (-0.0223 to -0.0023) | 0.0162 (0.0135 to 0.0189) | 0.0126 (0.0026 to 0.0226) | -0.0006 (-0.0022 to 0.001) |
| 60-69 | 0.0047 (-0.0019 to 0.0112) | -0.0116 (-0.0221 to -0.0011) | 0.0178 (0.015 to 0.0206) | 0.0136 (0.0031 to 0.0241) | 0.0007 (-0.0009 to 0.0024) |
| 70+ | 0.0122 (0.005 to 0.0193) | 0.0027 (-0.0088 to 0.0143) | 0.0202 (0.0171 to 0.0233) | 0.0397 (0.0282 to 0.0512) | 0.0009 (-0.001 to 0.0027) |
| Percent of female | 0.033 (0.0219 to 0.0442) | 0.0635 (0.0456 to 0.0814) | -0.0032 (-0.008 to 0.0017) | 0.0219 (0.004 to 0.0397) | -0.0036 (-0.0064 to -0.0007) |
| SDI index | 0 (0 to 0) | 0 (0 to 0) | 0 (0 to 0) | 0 (0 to 0) | 0 (0 to 0) |
| Unemployment rates | -0.9098 (-1.2049 to -0.6148) | -1.3307 (-1.8044 to -0.857) | 0.1566 (0.0283 to 0.2848) | 0.7536 (0.2814 to 1.2257) | 0.0913 (0.0157 to 0.1669) |
| PAF | -0.0008 (-0.0015 to -0.0001) | -0.0018 (-0.0029 to -0.0007) | 0.0002 (-0.0001 to 0.0005) | -0.002 (-0.0031 to -0.0009) | 0.0002 (0 to 0.0004) |

Abbreviation: SDI, socio-demographic Index; PAF, population attributable population.
